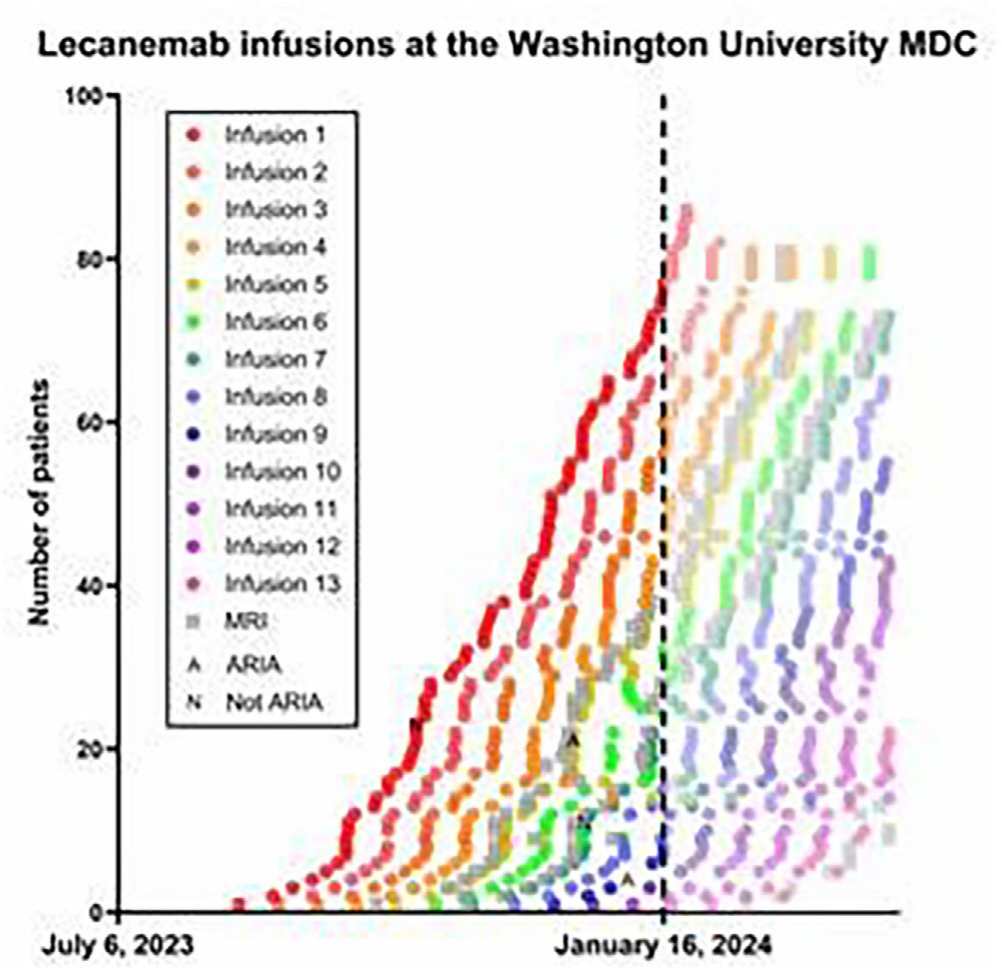# The first year of amyloid immunotherapy in an academic dementia specialty practice

**DOI:** 10.1002/alz.086217

**Published:** 2025-01-09

**Authors:** Madeline Paczynski, Suzanne E. Schindler, Erik S. Musiek, David M. Holtzman, Tammie L.S. Benzinger, Alan Dow, Sheyda J Namazie‐Kummer, Zachary J Posey, Carson Woodfin, Dawn Ellington, John C. Morris, B. Joy Snider

**Affiliations:** ^1^ Washington University School of Medicine, Saint Louis, MO USA; ^2^ Knight Alzheimer Disease Research Center, Saint Louis, MO USA; ^3^ Washington University in St. Louis School of Medicine, St. Louis, MO USA; ^4^ Knight Alzheimer Disease Research Center, St. Louis, MO USA; ^5^ Barnes Jewish Hospital, Saint Louis, MO USA; ^6^ Barnes Jewish Corporation, Saint Louis, MO USA; ^7^ Washington University in St. Louis, School of Medicine, St. Louis, MO USA

## Abstract

Implementation of amyloid‐lowering treatments in clinical care for early symptomatic Alzheimer disease (AD) raises many challenges. The Memory Diagnostic Center (MDC), the dementia specialty practice associated with Barnes‐Jewish Hospital/Washington University School of Medicine (BJH/WUSM), has 16 clinicians (12 physicians and 4 advanced practice providers) who see over 2,000 patients with memory disorders per year. BJH is the academic flagship of BJC HealthCare (BJC), an integrated health system in St. Louis, Missouri. Leadership of BJC/WUSM and MDC staff met regularly for two years in anticipation of amyloid‐lowering treatments becoming available. BJC/WUSM decided not to offer aducanumab (Aduhelm®) but did approve use of lecanemab (Leqembi®) after it received FDA approval in July 2023. Providing amyloid‐lowering treatments required coordination with BJC administration, neuroradiology, infusion centers and the clinical practice. Patients meeting appropriate use criteria were identified prior to approval of lecanemab. The requirement for AD biomarkers led to an increase in referrals to our lumbar puncture clinic prior to ∼30/month; there was less increase in referrals for AD blood tests or amyloid PET scans. The first patient received lecanemab within 6 weeks of FDA approval. Barriers to treatment included limited availability of appropriate MRI imaging, lack of reimbursement for AD blood tests and amyloid PET, limited capacity of infusion centers, and issues with private insurers. The MDC developed an on‐call system for response to infusion reactions and other adverse events and a tracking system for infusions and monitoring MRIs. We are developing registries and mechanisms in the electronic medical record to alert Emergency Department providers that patients on lecanemab being evaluated for stroke could be at increased risk of bleeding from thrombolytic therapies. Increasing capacity for patient assessments, biomarker testing, infusions, and follow‐up appointments has required adding advanced practice providers and office staff and presents challenges for reimbursement. As of January 2024, 80 MDC patients had received at least one lecanemab infusion and about 30 are undergoing evaluation to initiate treatment. Ongoing challenges include extending amyloid immunotherapy beyond the academic medical center, which will require training neurologists throughout the BJC system and developing sustainable staffing and billing models for management of amyloid‐lowering treatments.